# *Saccharomyces cerevisiae* metabolism in ecological context

**DOI:** 10.1093/femsyr/fow080

**Published:** 2016-09-14

**Authors:** Paula Jouhten, Olga Ponomarova, Ramon Gonzalez, Kiran R. Patil

**Affiliations:** 1 Structural and Computational Biology, European Molecular Biology Laboratory, Meyerhofstrasse 1, Heidelberg, DE 69117, Germany; 2 Department of Microbiologia, Instituto de Fermentaciones Industriales (CSIC), C. Juan de la Cierva 3, Madrid, ES 28006, Spain

**Keywords:** *Saccharomyces cerevisiae*, microbial community, genotype–phenotype relation, species interaction, ecological context

## Abstract

The architecture and regulation of *Saccharomyces cerevisiae* metabolic network are among the best studied owing to its widespread use in both basic research and industry. Yet, several recent studies have revealed notable limitations in explaining genotype–metabolic phenotype relations in this yeast, especially when concerning multiple genetic/environmental perturbations. Apparently unexpected genotype–phenotype relations may originate in the evolutionarily shaped cellular operating principles being hidden in common laboratory conditions. Predecessors of laboratory *S. cerevisiae* strains, the wild and the domesticated yeasts, have been evolutionarily shaped by highly variable environments, very distinct from laboratory conditions, and most interestingly by social life within microbial communities. Here we present a brief review of the genotypic and phenotypic peculiarities of *S. cerevisiae* in the context of its social lifestyle beyond laboratory environments. Accounting for this ecological context and the origin of the laboratory strains in experimental design and data analysis would be essential in improving the understanding of genotype–environment–phenotype relationships.

## Abstract (precis)

Could complex genotype–phenotype observations in *Saccharomyces cerevisiae* originate from its evolutionary history rich with social life?

## INTRODUCTION

In laboratories, *Saccharomyces cerevisiae* is usually grown in isolation and under well-defined conditions. Laboratory experiments therefore only faintly represent the challenges in the natural ecological habitats of wild and domesticated (i.e. strains adapted to human use for food and beverage fermentation already thousands of years ago) *S. cerevisiae*. Furthermore, in a natural habitat, the yeast metabolism needs to adapt and respond to the presence of other species. The absence of interspecies social life and unrepresentative growth conditions in laboratory experiments may thus hide the evolutionarily shaped operating principles of *S. cerevisiae* metabolic and regulatory networks. Furthermore, minimal spatial variation in liquid laboratory cultures hardly supports the phenotypic heterogeneity arising due to chemical gradients and physical proximity (Campbell *et al*. 2015; Campbell, Vowinckel and Ralser 2016). The laboratory studies of *S. cerevisiae*, with the above-mentioned limitations, are also generally limited to few strains. All these factors may have unforeseen and fundamental effects on the interpretation of experimental data and thereby present a challenge for building a quantitative understanding of the genotype–phenotype relationship.

### *Saccharomyces cerevisiae* metabolic responses are yet difficult to predict

Genome-scale metabolic models can be used to predict the phenotype dependence on the status of metabolic genes (Forster *et al*. 2003; Herrgard *et al*. 2008). These models are a gene-annotated stoichiometric representation of the full metabolic potential of a species augmented with thermodynamic and reaction capacity constraints. Despite the wealth of knowledge represented by these models, the prediction of gene essentiality is not yet flawless (Heavner and Price 2015), and the ability to predict the dependence of the metabolic flux distribution on the gene status (presence/absence) has been found to be poor (Pereira, Nielsen and Rocha 2016). In addition, the prediction accuracy of conditional gene essentialities in prototrophic deletion mutants on different carbon and nitrogen source has been found to be weak (VanderSluis *et al*. 2014). Furthermore, synthetic lethal phenotypes arising from genetic interactions can hardly be explained using metabolic models (Szappanos *et al*. 2011; Brochado *et al*. 2012). In auxotrophic laboratory strains grown on supplemented media, the interpretation of phenotypes is further complicated by yeast metabolizing the supplements, and the cessation of pathways because of the end product availability. This has shown to lead to complex molecular phenotypes through epistatic effects (Brem *et al*. 2002; Mulleder *et al*. 2012; Campbell *et al*. 2015).

### *Saccharomyces cerevisiae* laboratory genotypes

A majority of laboratory experiments are performed with only a few strains of *S. cerevisiae* which may not represent the full genetic potential of the species (Steinmetz *et al*. 2002; Carreto *et al*. 2008; Ehrenreich *et al*. 2010; Warringer *et al*. 2011; Strope *et al*. 2015). *S. cerevisiae* strains from genotypically different population origins exhibit large trait divergence in terms of growth characteristics on various substrates, in the presence of toxins or effectors, and mineral and vitamin limitations (Warringer *et al*. 2011). A laboratory strain may even lack evolutionary streamlining of the genotype–phenotype relation if its genome has not undergone the evolutionary selection under the corresponding growth conditions. Indeed, Qian *et al*. (2012) observed that in a common laboratory culture media, a laboratory strain of *S. cerevisiae* expresses genes that are rather deleterious than beneficial, indicating antagonistic pleiotropy that has not been resolved by adaptation to the corresponding environment (Qian *et al*. 2012). Relaxation of natural selection pressures has also been found to enrich, possibly through genetic drift, population specific alleles (Warringer *et al*. 2011; Zorgo *et al*. 2012). Additionally, auxotrophies commonly present in laboratory strains have been shown to affect the expression of a large number of genes and metabolite levels even when the growth medium is adequately supplemented (Brem *et al*. 2002; Mulleder *et al*. 2012). The supplementation thus causes effects beyond the stoichiometric fulfillment of the nutrients corresponding to the auxotrophies.

### Habitats of *Saccharomyces cerevisiae*

The ecology of the wild *S. cerevisiae* is relatively poorly understood (Boynton and Greig 2014), mainly because of early domestication (Sicard and Legras 2011) and widespread use of commodity strains. *S. cerevisiae* has been used for food and beverage fermentation for several thousand years due to its unique metabolic properties: fermentative metabolism, resistance to high sugar and ethanol concentrations, and production of specific aroma compounds. Humans have therefore significantly facilitated dispersal of the yeast (Goddard *et al*. 2010). For instance, the strains used for wine fermentation in Australia, Chile and New Zealand have shared recent ancestors with European wine strains (Legras *et al*. 2007; Liti *et al*. 2009; Goddard *et al*. 2010; Dunn *et al*. 2012). Through a large-scale population history study the genotypes of *S. cerevisiae* were found to fit to five primary lineages with shared ancestor populations (i.e. Malaysian, West African, North American, European and Sake) (Liti *et al*. 2009; Liti 2015). The genetic variations found in strains in a lineage were unique and equally distributed in the genome (Liti *et al*. 2009). However, a separate investigation of Chinese wild *S. cerevisiae* isolates revealed a larger and hitherto unknown reservoir of genetic variation (Wang *et al*. 2012). The natural history of *S. cerevisiae* including the known genetic variation is comprehensively reviewed by Liti (Liti 2015).

While *S. cerevisiae* is very abundant in human-made environments, such as wineries (Ciani *et al*. 2004), it appears to be rather rare in natural reservoirs (Goddard and Greig 2015). Thus, investigations of wild isolates are hindered by small population sizes (Liti 2015). In a search for the natural *S. cerevisiae* habitats, it has been isolated from plants (Wang *et al*. 2012), and the bark and leaves of oak trees and oak-associated soil (Sniegowski, Dombrowski and Fingerman 2002; Sampaio and Goncalves 2008; Zhang *et al*. 2010). Recently, Kowallik and Greig (2016) observed that yeast was more abundant in oak leaf litter than in the oak bark and that the leaf litter provides a refuge all year round (Kowallik and Greig 2016). Consistently, it has also been confirmed that *S. cerevisiae* can sporulate in soil and survive in this stress-resistant state until more nutritious conditions arise (Knight and Goddard 2016). *S. cerevisiae* indeed seems to respond to lignocellulosic solids from Birch tree by activating stress tolerance mechanisms—an observation that we suggest could be due to its evolutionary linkage to the bark niche (Koppram *et al*. 2016). Despite the common belief that yeasts are naturally found on the surface of grapes, a study finds that only one in thousand grapes are positive for *S. cerevisiae* (Mortimer and Polsinelli 1999). In cases of damaged fruit or berries, on the other hand, the occurrence and cell counts of *S. cerevisiae* were found to be higher (Mortimer and Polsinelli 1999). Interestingly, insects serve also as natural reservoirs and vectors that promote yeast dispersal: *S. cerevisiae* can be found associated with flies (Chandler, Eisen and Kopp 2012), social wasps (Stefanini *et al*. 2012) and bees (Goddard *et al*. 2010). Given the hitherto focus on prokaryotes in microbial diversity analysis due to sample preparation constraints, further natural reservoirs of the yeast might not have been discovered yet.

The known natural reservoirs of *S. cerevisiae* are usually nutrient poor with occasional periods of rich resource availability (e.g. after a transfer from oak bark to a faulty fruit by an insect) (Liti 2015). Therefore, unlike human-associated yeasts, wild strains most likely spend the most of their life in a dormant state. It has been argued that *S. cerevisiae* does not show adaptations to any particular habitat, but rather an ability to survive in a wide range of conditions (such as temperature, pH, nutrient concentrations and osmolarity) (Goddard and Greig 2015). The tolerance to a variety of environmental perturbations is consistent with the lifestyle of nomadic generalist that inhabits diverse niches at low abundance. High adaptability of yeast is supported by a remarkable chromosomal number plasticity (Pavelka *et al*. 2010; Liu *et al*. 2015; Selmecki *et al*. 2015). Furthermore, yeast is capable of sexual reproduction, which also facilitates rapid adaptation (Goddard 2016). Nevertheless, variation in the natural ecological niches is shown to be reflected in trait divergence within *S. cerevisiae* strains associated with different population origins (Warringer *et al*. 2011).

Upon sudden exposure to excess glucose, even under aerobic conditions, *S. cerevisiae* exhibits high glycolytic and fermentative fluxes (Pronk, Steensma and vanDijken 1996)—a complex trait called short-term Crabtree effect. Several traits that contribute to the short-term Crabtree effect have appeared along the evolutionary history of *Saccharomyceta* (Hagman *et al*. 2013). Thus, *S. cerevisiae* exhibits an evolutionarily shaped trait to tolerate or even benefit from a sudden change in glucose availability.

In contrast to the natural reservoirs, usual laboratory growth medium is either a defined medium optimized for short generation times or a rich medium like in food and beverage fermentation applications of *S. cerevisiae*. Thus, the metabolism of *S. cerevisiae* is best understood in the fast growing states of fermentation. Wild strains from natural environments generally show lower glucose utilization rate than the domesticated strains of *S. cerevisiae* that have been selected in conditions of high glucose availability (Spor *et al*. 2009). Modeling results by Nidelet *et al*. (2016) accordingly suggest that the intracellular metabolic fluxes vary among *S. cerevisiae* strains from different ecological origins (bread, rum, wine, flour, Mediterranean and American oak) (Nidelet *et al*. 2016). Their results suggest that while the glycolytic and fermentative fluxes are similar between strains, the flux through the pentose phosphate pathway is strain dependent. Surprisingly, the domesticated (i.e. for food and beverage fermentation) strains show generally higher phenotypic diversity, in terms of growth characteristics under different environmental conditions, than the investigated wild strains of *S. cerevisiae*, even though the wild strains are genetically more diverse and show stronger geographical patterning (Warringer *et al*. 2011). The phenotypic diversity of the domesticated strains may result from selection for traits associated with specific fermentation substrates (e.g. rice fermentation for sake, grape for wine and barley for beer) (Liti *et al*. 2009; Boynton and Greig 2014; Gallone *et al*. 2016). For example, wine fermentations present challenging conditions such as high sugar concentration and low pH. Due to the equal abundance of glucose and fructose in the grape must, the total sugar concentration is extremely high creating challenging conditions of high osmolarity. The low pH of grape must originates from the presence of organic acids. When exposed to wine fermentation mimicking conditions, *S. cerevisiae* strains from different sources (i.e. laboratory strains, wild strains, clinical isolates, vineyard isolates, bakery strains, commercial wine strains, strains domesticated for other fermentation processes) showed distinct fermentation characteristics (Camarasa *et al*. 2011). While commercial wine yeasts were able to perform as they have been selected to, i.e. to complete the fermentation, strains from other sources commonly grew poorly and could not ferment the sugars completely. Notably, under the challenging conditions laboratory strains diverged from all the rest of the strains in their poor biomass formation and fermentation capability and in their product profile. Furthermore, since wine fermentations are commonly unsterilized, the domesticated *S. cerevisiae* wine strains have been simultaneously exposed to the extreme abiotic conditions in grape must and challenged with social life with other species.

### Social life of *Saccharomyces cerevisiae*—symbionts impact yeast metabolism

**Figure 1.**
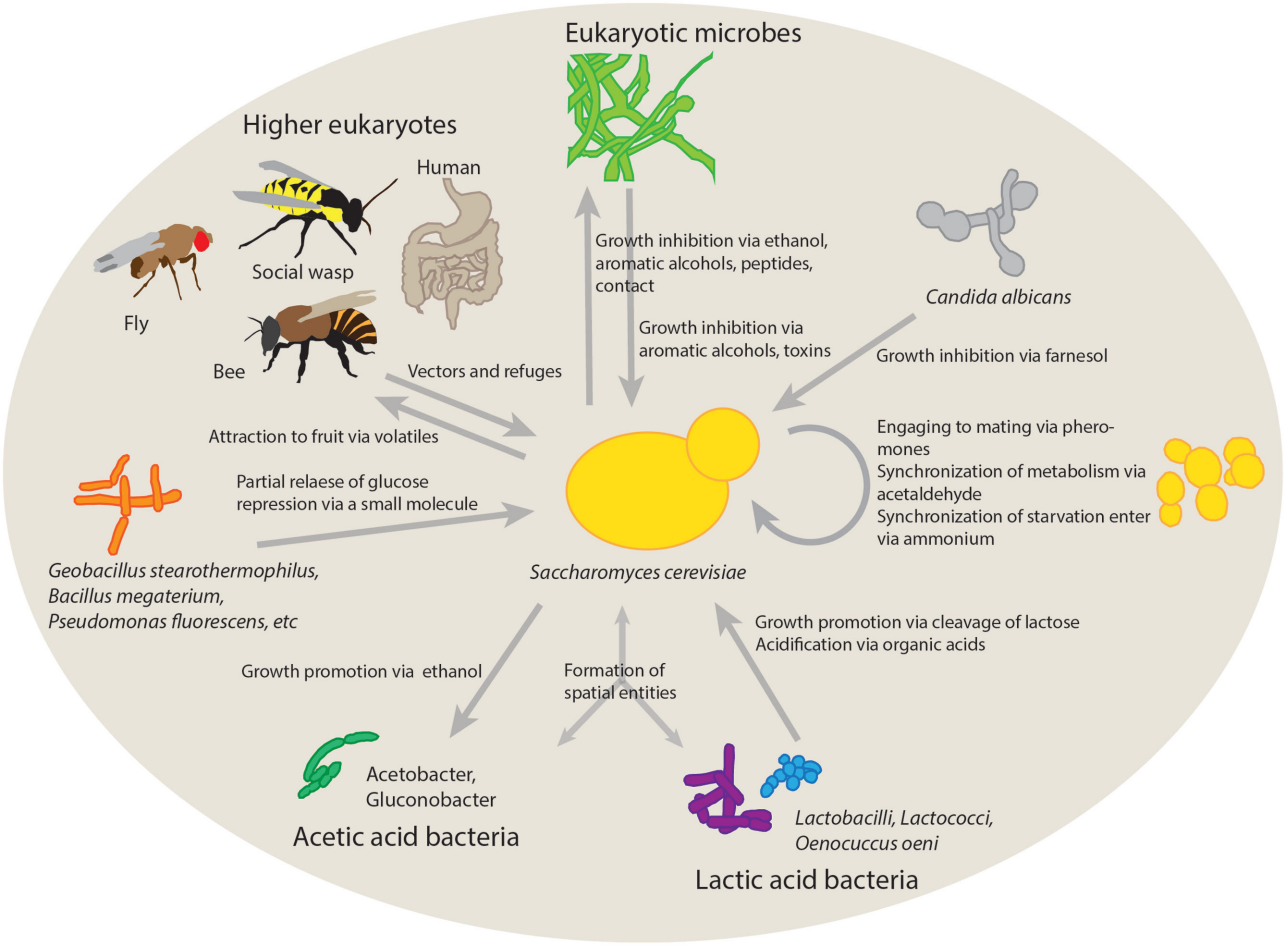
The social life of *S. cerevisiae.* Yeast shows numerous interactions with bacteria, other fungi and higher eukaryotes. Niche engineering is performed by the yeast and interacting organisms. *S. cerevisiae* cells communicate also within species by modifying the chemical environment. Yeast–plant interactions have not been considered here.

In addition to a dynamic abiotic environment, co-habiting organisms constitute another important ecological dimension shaping *S. cerevisiae* metabolism (Fig. 1). This social dimension also applies, despite being removed from their original ecological context, to domesticated yeasts growing in human created/controlled environments. Wine fermentation is perhaps the best-studied environment of *S. cerevisiae* with respect to the interspecies interactions (Mortimer and Polsinelli 1999; Fleet 2003; Alexandre *et al*. 2004; Comitini *et al*. 2005; Ciani *et al*. 2010; Barata, Malfeito-Ferreira and Loureiro 2012; Branco *et al*. 2014; Jolly, Varela and Pretorius 2014; Ramirez *et al*. 2015; Ciani *et al*. 2016; Liu *et al*. 2016; Wang, Mas and Esteve-Zarzoso 2016). Since grape must is typically not sterilized for wine fermentations, they represent a multispecies ecosystem. The yeast spectrum in the ecosystems includes over 40 different species that have been isolated from grape must (Jolly, Varela and Pretorius 2014). Understanding the interactions between fermenting yeasts and other microorganisms in wine has a distinct economic and gustatory incentive (Fleet 2003). More recently, social life of yeast has been observed also in other environments such as kefir (i.e. fermented milk product with originating from Caucasian mountains) (Simova *et al*. 2002; Farnworth 2005), sourdough (De Vuyst *et al*. 2014) and biofuel production cultures (Watanabe, Nakamura and Shima 2008; Lucena *et al*. 2010; Tiukova, Eberhard and Passoth 2014). Lactic acid bacteria (LAB) are commonly found together with *S. cerevisiae* in the domestic applications (e.g. wine, sourdough and kefir), but also in nature in overripened or faulty fruits (Barata, Malfeito-Ferreira and Loureiro 2012). Furthermore, LAB are common spoilage organisms in open *S. cerevisiae* fermentations used for biofuel production (Watanabe, Nakamura and Shima 2008; Lucena *et al*. 2010; Tiukova, Eberhard and Passoth 2014). In bioethanol production, *Lactobacillus fermentum* and *L. brevis* have been reported as the most common contaminants (Watanabe, Nakamura and Shima 2008). Tiukova, Eberhard and Passoth (2014) observed that *L. vini* in co-cultures with *S. cerevisiae* or *Dekkera bruxellensis* causes bacteria–yeast aggregation, thereby decreasing the ethanol production by yeast (Tiukova, Eberhard and Passoth 2014). *S. cerevisiae* can in turn benefit from the presence of LAB, for example, in lactose-rich environments. While *S. cerevisiae* is not able to utilize lactose, LAB are able to cleave it into glucose and galactose, which can be readily metabolized by the yeast (Mendes *et al*. 2013) (Fig. 1). Organic acids produced by fermentative microbes may also benefit yeast. *S. cerevisiae* is capable of consuming lactate during cheese ripening, simultaneously producing valuable aroma compounds (Kagkli *et al*. 2006). Though active transport of other acids such as malate and succinate across the cell membrane is not known in *S. cerevisiae*, these may diffuse into the cell contributing to the metabolism (Barnett and Kornberg 1960). The laboratory strains of *S. cerevisiae* show specific regulatory responses towards weak acids (Mira, Teixeira and Sa-Correia 2010), but the dependence of this response on the natural habitat or the origin of *S. cerevisiae* strains is yet unexplored.

In addition to organic acids, other compounds secreted by coexisting species may affect *S. cerevisiae* phenotypes by directly triggering regulatory networks. Recently, some bacteria were found to partially release glucose repression in *S. cerevisiae* by inducing a prion-like element through secretion of a yet unidentified small molecule(Jarosz *et al*. 2014a,b) (Fig. 1). Several different bacteria were shown to relieve glucose repression when co-cultured with *S. cerevisiae*. This was found to benefit both the bacterial and yeast symbionts. While lower ethanol production reduced the toxic effect on bacteria, the yeast gained an ability to utilize other carbon sources, which was found particularly advantageous in mixed carbon source media (e.g. molasses).

Fungal symbionts of yeast can also impact its growth and metabolism through toxins and signaling molecules (Fig. 1). For instance, a toxin-secreting *Torulaspora delbrueckii* strain is able to kill *S. cerevisiae* (Ramirez *et al*. 2015). Another example is growth impairment of *S. cerevisiae* by *Candida albicans* through secretion of farnesol, a quorum-sensing molecule (Machida *et al*. 1998). Farnesol is an isoprenoid alcohol that interferes with the cell cycle signaling in *S. cerevisiae* in particular, but also affects the mitochondrial function through increased generation of reactive oxygen species (Machida *et al*. 1998, 1999). Aromatic alcohols tyrosol, tryptophol and phenylethanol are further fungal quorum-sensing molecules, which *S. cerevisiae* is also able to produce (Bruce *et al*. 2004) and can induce changes in growth morphology (Cottier and Muhlschlegel 2012).

Certain volatiles produced by yeast, commonly higher alcohols and their esters, attract flies, social wasps and bees. These insects can thereby transport *S. cerevisiae* to new habitats (Chandler, Eisen and Kopp 2012) (Fig. 1). Yeasts in turn can benefit its host through establishing a mutualistic interaction. While *S. cerevisiae* that attract flies better get an advantage of effective dispersal (Gilbert 1980; Chandler, Eisen and Kopp 2012; Hoang, Kopp and Chandler 2015) and outbreeding (Reuter, Bell and Greig 2007), *Drosophila* populated with more attractive yeast species demonstrate higher fecundity (Buser *et al*. 2014). Larvae of flies also benefit from yeast symbiont, mainly as a dietary supplement facilitating development and survival (Anagnostou, Dorsch and Rohlfs 2010).

### Social life of *Saccharomyces cerevisiae*—yeast impacts community composition

Metabolites and peptides secreted by yeast can have a substantial impact on its co-habitants. Metabolites produced by *S. cerevisiae* have been found to reduce the cultivability of a number of non-*Saccharomyces* yeasts during co-fermentation or in conditioned medium (Wang, Mas and Esteve-Zarzoso 2016). *S. cerevisiae* produces high concentrations of ethanol that are toxic for many other microbial species. *Saccharomyceta* have gained traits contributing to the high fermentative capacity along their evolutionary history (Hagman *et al*. 2013). Only more recently *S. cerevisiae* with its closely related species gained a further increased ethanol production capability through a trait in which respiration becomes repressed in a high glucose environment even when oxygen is available (i.e. long-term Crabtree effect; Pronk, Steensma and vanDijken 1996) (Hagman and Piskur 2015). The high fermentative capacity allows to convert sugar to ethanol at a fast rate, and later shift to a respiratory metabolic phenotype to consume the ethanol (diauxic shift). The yeast thus seems to apply a make-accumulate-consume strategy in high glucose environments (Piskur *et al*. 2006).

While ethanol has considered to be the toxic product which *S. cerevisiae* benefits from the most, it has recently become evident that it may not be the most efficient of the weapons of *S. cerevisiae*. *S. cerevisiae* employs a 2-fold strategies to inhibit the growth of bacteria and fungi: secretion of small molecules and proteinaceous compounds such as peptides and physical cell–cell contact (Nissen, Nielsen and Arneborg 2003; Piskur *et al*. 2006; Branco *et al*. 2014; Wang, Mas and Esteve-Zarzoso 2016) (Fig. 1). In addition to ethanol, *S. cerevisiae* produces volatile compounds such as aromatic alcohols, which are implicated in inhibition of other fungi (Bruce *et al*. 2004; Cottier and Muhlschlegel 2012). *S. cerevisiae* secretes antimicrobial peptides that have either fungistatic (e.g. against *Lachanchea thermotolerans* (*Kluyveromyces thermotolerans*) and *T. delbrueckii*) or even fungisidic (e.g. against *Kluyveromyces marxianus*) effects on other fungal microbes (Albergaria *et al*. 2010). When exposed to *S. cerevisiae*'s antimicrobial peptides, *Hanseniaspora guilliermondii* suffers from alterations in membrane permeability leading to a severe loss of intracellular pH homeostasis (Branco *et al*. 2015). Interestingly, it was recently found that anti-fungal and anti-bacterial killer peptides secreted by *S. cerevisiae* during wine fermentation include peptides of glyceraldehyde dehydrogenase (GAPDH), a glycolytic enzyme (Branco *et al*. 2014). The polypeptides of the GAPDH isoenzymes have been observed to be associated also with the cell wall (Delgado *et al*. 2001) where they are amenable for interactions with other species.

Cell–cell contact with *S. cerevisiae* has been found to contribute to the death of *La. thermotolerans* (*K. thermotolerans*) and *T. delbrueckii* in wine fermentations (Nissen, Nielsen and Arneborg 2003; Kemsawasd *et al*. 2015). This phenomenon could possibly be mediated by the anti-fungal peptides residing attached to the cell membrane.

Besides producing toxic compounds to fight competitors, *S. cerevisiae* can also provide benefits to other species. Already in 1965, commensalism of bacterium *Proteus vulgaris* with *S. cerevisiae* was observed (Shindala *et al*. 1965). The growth benefit of co-culturing with the yeast was found dependent on a niacin-like nutrient secreted by *S. cerevisiae* and essential for *P. vulgaris*. Megee *et al*. (1972) reported that they had been able to create various relationships (i.e. commensalism, competition and mutualism) between *S. cerevisiae* and *Lactobacillus casei* by varying the concentrations of glucose and riboflavin in the medium (Megee *et al*. 1972).

The stationary state survival of *Pseudomonas putida* increases substantially in co-culture with *S. cerevisiae*, which consumes glucose rapidly and lowers the pH (Romano and Kolter 2005). *Saccharomyces cerevisiae* has also been shown to support the growth of *L. delbrueckii* ssp. *bulgaricus* in lactose media by supplying L-Alanine and CO_2_ to the bacteria (Mendes *et al*. 2013). In return, the bacteria provided galactose, as a cleavage product of lactose, for *S. cerevisiae* growth.

In the late stages of wine fermentation, yeast can either promote or inhibit the growth of LAB involved in malolactic fermentation (Liu *et al*. 2016). The yeast inhibiting bacteria secretes sulfur-containing peptides, in distinction to the yeast phenotype creating a supporting niche for LAB. Together with LAB, acetic acid bacteria (AAB) commonly co-occur with *S. cerevisiae* (Farnworth 2005; Camu *et al*. 2007). *Acetobacter, Gluconobacter* and *Gluconacetobacter* species of AAB are the most prevalent co-occurring taxons. *S. cerevisiae* together with LAB and AAB species has been found for instance in spontaneous cocoa bean (Camu *et al*. 2007), kefir (Farnworth 2005) and kombucha (Jayabalan *et al*. 2014) fermentations. In spontaneous cocoa bean fermentations, yeasts, including *S. cerevisiae*, together with LAB engineer a niche for AAB. LAB consume citrate initially present, thus, increasing pH that favors AAB. Mainly *Acetobacter pasteurianus* oxidizes ethanol produced by the yeasts (Fig. 1). Oxygen-dependent conversion of ethanol to acetate and/or acetoin increases the temperature until the microbial activities cease. Ethanol and acetate diffuse into the cocoa beans creating the desired flavor and color characteristics. In addition to ethanol, AAB oxidize lactic acid secreted by LAB and are also able to oxidize glucose to gluconic acid and mannitol into fructose, at least in the presence of ethanol (Moens, Lefeber and De Vuyst 2014). Thus, ethanol produced by yeast enables AAB to oxidize a wider range of substrates.

### Phenotypic heterogeneity and cross-feeding in yeast populations

In a yeast colony, the cells encounter a variable chemical environment depending on their location. This may induce phenotypic heterogeneity within species, possibly enhanced by intraspecies communication by secreted metabolites. While complementary auxotrophic strains of *S. cerevisiae* have been found to fail to cross-feed sufficiently for survival, a viable complementary cross-feeding community is formed when the auxotrophs emerge from a self-supporting cell during colony formation (Campbell *et al*. 2015; Campbell, Vowinckel and Ralser 2016). This phenomenon was discovered in a system where the cells carried in separate plasmids the genes for the synthesis of four compounds essential for growth (i.e. histidine, leucine, uracil and methionine). During colony development, prototrophy was progressively lost leading to phenotypic heterogeneity, but the community could survive by exchanging the essential nutrients.

In colony development, *S. cerevisiae* cells behave periodically with acidic and alkali phases (Palkova *et al*. 1997). When the cells switch from an acidic phase to an alkaline phase, they produce volatile ammonia as a pulse, which triggers ammonia production in surrounding colonies. The released ammonia signals for the synchronization of nutrient starvation response. Ammonium secreted by *S. cerevisiae* could potentially be sensed by other yeasts as well (Gori *et al*. 2007). Other small molecules can also mediate communication between yeast cells (Fig. 1). At high cell densities in liquid cultures, *S. cerevisiae* cells synchronize their metabolism with secreted acetaldehyde (Richard *et al*. 1996). Other fungi can thus also influence *S. cerevisiae* metabolism through acetaldehyde (Cheraiti, Guezenec and Salmon 2005). Other examples of communication include bicarbonate for synchronization of the onset of sporulation or meiosis (Hayashi, Ohkuni and Yamashita 1998; Ohkuni, Hayashi and Yamashita 1998), small peptide pheromones used to facilitate partner finding during sexual reproduction (Jones and Bennett 2011) and secreted Hsp12p protein as a ‘danger signal’ in order to activate the stress response in the surrounding yeast cells (Rivero *et al*. 2015).

## CONCLUSIONS

Most of the current knowledge of *S. cerevisiae* metabolism pertains to (or is interpreted in the context of) its life as a single entity, devoid of species interactions, and in a limited set of laboratory conditions. The limited understanding of the challenges and possibilities that have evolutionarily shaped the metabolic and regulatory systems of yeast may be the major factor hindering us from explaining complex genotype–environment–phenotype interactions. Accounting for the ecological context of yeast could also help us to assign functions to the uncharacterized 10% of genes (669 uncharacterized open reading frames, www.yeastgenome.org, on 15 August 2016) in the yeast genome, and to identify moonlighting functions of metabolic enzymes. Mainly pioneered by enological research, phenotypic peculiarities arising from the social life of yeast are increasingly being revealed. These findings present an excellent opportunity towards building more accurate models of metabolic and regulatory networks. The full phenotypic potential to be revealed will also increase the application possibilities of the already widely used industrial production host.
